# The clinical utility of routine spinal radiographs by chiropractors: a rapid review of the literature

**DOI:** 10.1186/s12998-020-00323-8

**Published:** 2020-07-09

**Authors:** Melissa Corso, Carol Cancelliere, Silvano Mior, Varsha Kumar, Ali Smith, Pierre Côté

**Affiliations:** 1 Faculty of Health Sciences, Ontario Tech University and Centre for Disability Prevention and Rehabilitation, 2000 Simcoe St N, Oshawa, ON L1G 0C5 Canada; 2 Canadian Memorial Chiropractic College, North York, Ontario Canada

**Keywords:** Spine, Radiograph, X-ray, Clinical utility, Chiropractor, Posture analysis

## Abstract

**Introduction:**

When indicated by signs or symptoms of potentially serious underlying pathology (red flags), chiropractors can use radiographs to inform their diagnosis. In the absence of red flags, the clinical utility of routine or repeat radiographs to assess the structure and function of the spine is controversial.

**Objectives:**

To determine the diagnostic and therapeutic utility of routine or repeat radiographs (in the absence of red flags) of the cervical, thoracic or lumbar spine for the functional or structural evaluation of the spine. Investigate whether functional or structural findings on repeat radiographs are valid markers of clinically meaningful outcomes. The research objectives required that we determine the validity, diagnostic accuracy and reliability of radiographs for the structural and functional evaluation of the spine.

**Evidence review:**

We searched MEDLINE, CINAHL, and Index to Chiropractic Literature from inception to November 25, 2019. We used rapid review methodology recommended by the World Health Organization. Eligible studies (cross-sectional, case-control, cohort, randomized controlled trials, diagnostic and reliability) were critically appraised. Studies of acceptable quality were included in our synthesis. The lead author extracted data and a second reviewer independently validated the data extraction. We conducted a qualitative synthesis of the evidence.

**Findings:**

We identified 959 citations, screened 176 full text articles and critically appraised 23. No relevant studies assessed the clinical utility of routine or repeat radiographs (in the absence of red flags) of the cervical, thoracic or lumbar spine for the functional or structural evaluation of the spine. No studies investigated whether functional or structural findings on repeat radiographs are valid markers of clinically meaningful outcomes. Nine low risk of bias studies investigated the validity (*n* = 2) and reliability (*n* = 8) of routine or repeat radiographs. These studies provide no evidence of clinical utility.

**Conclusion:**

We found no evidence that the use of routine or repeat radiographs to assess the function or structure of the spine, in the absence of red flags, improves clinical outcomes and benefits patients. Given the inherent risks of ionizing radiation, we recommend that chiropractors do not use radiographs for the routine and repeat evaluation of the structure and function of the spine.

## Introduction

In the United States in 2010, the rate of spine radiographs within 5 days of presenting to a chiropractor was 204 per 1000 new patients [1]. An analysis of national trends in the United States suggests that the rate of spinal radiography by chiropractors and podiatrists increased by 14.4% between 2003 and 2015 [2]. This increase occurred despite the publication of several evidence-based clinical practice guidelines and clinical prediction rules to assist chiropractors in determining the indication for spine radiographs to assist with diagnosing a pathology [3–7]. Overall, guidelines suggest that radiographs are indicated when signs and symptoms of potentially serious underlying pathology (red flags) are identified through the clinical history and physical examination. However, on its own, an isolated “red flag” may have a high false positive rate for the diagnosis of underlying spinal pathology, such as cancer [8]. For example, the presence of a solitary “red flag” such as age over 50 years may not be sufficient to warrant taking spine radiographs [8, 9]. Therefore, clinicians are encouraged to combine sound clinical judgement and the assessment of red flags when ordering radiographs [9–11].

In the absence of “red flags”, the use of spinal radiographs is not recommended [3–7]. Nevertheless, factions of chiropractors, including the International Chiropractic Association promote the use of routine or repeat radiographs to assess the structure and function of the spine [12–14]. This practice which dates back to 1910 was initiated when no evidence was available to guide the judicious use of spine radiographs [15]. Historically, these groups of chiropractors have argued that radiographs are helpful to measure postural abnormalities, identify vertebral misalignment or subluxation and guide treatment with spinal manipulative therapy [12, 15, 16]. The belief that radiographs are useful to detect and correct spine structure and function provides the foundation for many chiropractic technique systems that are still in use today. To our knowledge, approximately 23 chiropractic techniques use spine radiography (including full spine radiography) to guide the clinical management of patients [16]. These include the Gonstead, Chiropractic BioPhysics®, Toggle-Recoil, and National Upper Cervical Chiropractic Association (NUCCA) techniques [16]. Proponents of these techniques claim that the use of routine and repeat radiographs is supported by scientific evidence and have published a guideline to assist clinicians with the biomechanical assessment of spinal subluxation in chiropractic clinical practice using radiography [13]. However, these claims have not yet been evaluated for their clinical utility, the benefit a patient gains from a test or treatment [17–19]. This was a particular concern for the College of Chiropractors of British Columbia (CCBC) which regulates the practice of chiropractic in the province of British Columbia, Canada. The mission of the CCBC is to protect the public by regulating British Columbia’s doctors of chiropractic to ensure safe, qualified and ethical delivery of care [20].

At the request of the CCBC, we conducted an independent rapid review of the literature to investigate the clinical utility of routine and repeat radiographs (in the absence of red flags) for the structural and functional evaluation of the spine by chiropractors. Specifically, we aimed to investigate: 1) the diagnostic utility of radiographs of the cervical, thoracic or lumbar region for the structural and functional evaluation of the spine; 2) the therapeutic utility of radiographs of the cervical, thoracic or lumbar region for the structural and functional evaluation of the spine; and 3) whether functional or structural findings on repeat radiographs of the cervical, thoracic or lumbar spine are valid markers of clinically meaningful change when monitoring conditions or managing patients. Our three main research objectives required that we first determine the validity and reliability of radiographs for the structural and functional evaluation of the spine.

## Methods

We conducted a rapid review of the literature. Rapid reviews are used by health decision-makers (clinicians, patients, managers, and policy makers) who need timely access to health information to plan, develop and implement health care and policies [21, 22]. We used methodology recommended by the World Health Organization to answer our questions and previously used by our group [21, 23].

### Protocol and registration

We reported our review according to the Preferred Reporting Items for Systematic Reviews and Meta-Analysis (PRISMA) and PRISMA Harms checklists [24, 25]. We registered our review with the International Prospective Register of Systematic Reviews (PROSPERO) on November 12, 2019 (CRD42020158321).

### Clinical utility

**Fig. 1:**
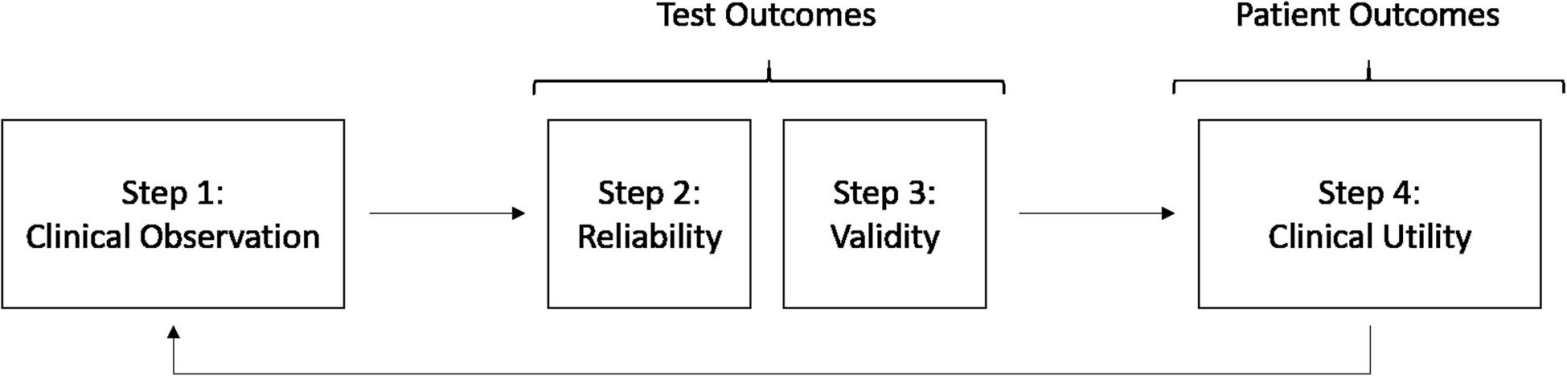
Flow of investigations leading to the determination of the clinical utility of a test

Clinical utility is defined as the benefit that a person has from an intervention or test [17]. Clinical utility includes diagnostic utility (the degree to which the use of a test is associated with changing health outcomes) [18] and therapeutic utility (the degree to which a test contributes to improving health outcomes through the selection of an appropriate treatment) [19]. Demonstrating that a test has clinical utility requires demonstration that patients benefit from a test in a well-designed randomized clinical trial (RCT) or cohort study [17]. However, preliminary steps are necessary before the hypothesis that a clinical test has clinical utility can be tested (Fig. 1). First, the hypothesis that a test (e.g. spine radiographs) may benefit patient care must be generated from sound clinical observations. Second, the validity, diagnostic accuracy and reliability of the clinical test must be investigated [26–28]. Studies of diagnostic accuracy should report the sensitivity, specificity, predictive values and likelihood ratios of the test under investigation [26, 27] [Additional file 2]. Tests that are not valid, reliable or lack diagnostic accuracy are unlikely to have clinical utility, and therefore, unlikely to benefit patients [17–19]. Our methodology includes an evaluation of the diagnostic accuracy and reliability of radiographs used to evaluate the structure and function of the spine by chiropractors. Finally, tests that are reliable, valid and have diagnostic accuracy must demonstrate clinical (i.e., diagnostic and therapeutic) utility, in other words, impact health outcomes.

### Eligibility criteria

#### Participants and interventions

We included studies of patients presenting to chiropractors who received spinal radiographs of the cervical, thoracic or lumbar region, in the absence of red flags.

#### Comparators

We considered comparisons with participants who did not receive spinal radiographs or were assessed with other spinal examination methods, such as palpation, postural evaluation or other diagnostic imaging techniques (such as CT scan or MRI).

#### Outcomes

We investigated structural or functional outcomes associated with various chiropractic approaches that use radiographs as diagnostic or assessment tools. Such approaches may include assessing for asymmetry in vertebral alignment as measured by line drawings, spinal curvatures, and the presence and correction of vertebral dysfunction as determined by measurement or positional listings. We also considered patient important outcomes throughout a course of treatment, including but not limited to pain, functioning, self-reported recovery, health-related quality of life, or well-being.

#### Study designs

We included RCTs, cohort studies, case-control studies, cross-sectional studies, and diagnostic and reliability studies. We excluded guidelines, letters, editorials, commentaries, unpublished manuscripts, dissertations, government reports, books and book chapters, conference proceedings, meeting abstracts, lectures and addresses, consensus development statements, guideline statements, cadaveric, laboratory or animal studies, qualitative studies, systematic reviews and meta-analyses.

### Information sources

We developed our search strategy in consultation with a health sciences librarian, and a second librarian reviewed the strategy to ensure accuracy. We systematically searched three databases that thoroughly index the manual therapy literature published by various health professions from inception to November 25, 2019: MEDLINE (U.S. National Library of Medicine, through Ovid Technologies Inc.), Cumulative Index to Nursing and Allied Health (CINAHL, through EBSCO*host*), and Index to Chiropractic Literature (ICL, Chiropractic Library Collaboration). Search terms consisted of subject headings specific to each database (e.g., MeSH in MEDLINE) and free text words relevant to our objectives and study design [see Additional file 1]. We restricted our search to papers published in English.

### Study selection

We used a two-phase screening process to identify eligible studies. In phase one screening, we reviewed titles and abstracts and classified articles as possibly relevant or irrelevant. During phase two screening, we reviewed the full text of possibly relevant articles for final determination of eligibility.

A trained investigator (MC) conducted all of the screening. Prior to phase one and phase two screening, we validated the quality of screening by MC. Ten percent of all eligible articles were randomly selected and the titles and abstracts (phase one) and full text (phase two) of these articles were screened independently by a second experienced investigator (CC). A 95% level of agreement was required between two reviewers before moving to full screening. Once the 95% agreement was achieved, one reviewer (MC) completed phase one and two screening.

### Risk of Bias in individual studies

The lead author (MC) critically appraised the internal validity of relevant articles using the Scottish Intercollegiate Guidelines Network (SIGN) criteria for RCTs, cohort studies and case-control studies [29, 30], a checklist created by Hoy et al. for cross-sectional studies [31], the Quality Assessment of Diagnostic Accuracy Studies (QUADAS) for diagnostic studies [32] and the Quality Appraisal tool for studies of diagnostic Reliability (QAREL) for reliability studies [33].

We included a quality control step in the critical appraisal of studies. The investigator who assessed the risk of bias of the studies (MC) presented a summary of the critically appraised papers to four experienced methodologists (PC, SM, CC, VK) who validated the outcome of the appraisals. Disagreements regarding the internal validity of papers were resolved through discussion. The lead author created risk of bias tables for all eligible studies (Tables 1 and 2), which were validated by the other investigators (PC, SM, CC, VK). Studies were rated low risk of bias or at risk of bias.

**Table 1:** Risk of Bias Tables

Risk of Bias table: Diagnostic Studies																				
Author, Year	Phase	1.1	1.2	1.3	R	C	2.1	2.2	R	C	3.1	3.2	R	C	4.1	4.2	4.3	4.4	R	Overall Ax
Gregory, 1998 [34]	1	Y	N/A	N	H	L	Y	N/A	L	L	CS	Y	U	L	N/A	Y	Y	Y	L	At Risk of Bias
Harrison, 2002 [35]	1	CS	N/A	Y	U	L	CS	Y	U	L	Y	CS	U	L	N/A	Y	Y	Y	L	At Risk of Bias
Frymoyer, 1986 [36]	1	Y	N/A	CS	L	L	Y	CS	U	L	CS	Y	U	L	N/A	Y	Y	Y	L	At Risk of Bias
Harrison, 2003 [37]	1	CS	N/A	Y	U	L	CS	Y	U	L	Y	CS	U	L	N/A	Y	Y	Y	L	At Risk of Bias
Wight, 1999 [38]	1	CS	N/A	Y	U	L	CS	N/A	U	L	Y	CS	U	L	N/A	Y	Y	Y	L	At Risk of Bias
Rosok, 1993 [39]	1	Y	N/A	CS	L	L	N	N/A	U	L	Y	Y	L	L	N/A	Y	Y	CS	L	At Risk of Bias
Haas, 1992 [40]	1	CS	N/A	N	H	L	CS	N/A	U	L	Y	Y	L	L	N/A	Y	Y	Y	L	At Risk of Bias
Haas, 1992 [41]	1	CS	N/A	N	H	L	CS	N/A	U	L	Y	Y	L	L	N/A	Y	Y	Y	L	At Risk of Bias
Leboeuf, 1989 [42]	1	Y	N/A	Y	L	L	Y	Y	L	L	Y	CS	U	L	N/A	Y	Y	Y	L	At Risk of Bias
Phillips, 1986 [43]	1	Y	N/A	CS	L	L	CS	N/A	U	L	CS	Y	U	L	N/A	Y	Y	Y	L	At Risk of Bias
Rudy, 2015 [44]	2	N	N/A	Y	U	L	Y	Y	L	L	CS	Y	H	L	N/A	Y	Y	Y	L	At Risk of Bias
McAviney, [45]	2	Y	Y	N	L	L	Y	Y	L	L	Y	Y	L	L	N/A	Y	Y	Y	L	Low Risk of Bias
McGregor, 1995 [46]	2	Y	Y	CS	L	L	Y	N/A	L	L	CS	Y	L	L	Y	Y	Y	Y	L	Low Risk of Bias

*Y* yes; *N* no; *CS* Can’t Say; *H* high; *L* low; *N/A* not applicable; *U* Unclear; *R* Risk; *C* Concern

Legend: Diagnostic Studies, 1.1 Consecutive or Random Sample of Patients, 1.2 Case-control Design Avoided, 1.3 Avoid Inappropriate Exclusions, 2.1 Blinded Index Test Interpretation, 2.2 If Threshold Used, Pre-specified, 3.1 Reference Standard Classifies Condition, 3.2 Blinded Reference Test Interpretation, 4.1 Appropriate Interval Between Tests, 4.2 All Receive Reference Standard, 4.3 All Receive Same Reference Standard, 4.4 All Patients Included in Analysis

**Table 2:** Risk of Bias Tables

Risk of Bias table: Reliability Studies													
Author, Year	1.1	1.2	1.3	1.4	1.5	1.6	1.7	1.8	1.9	1.10	1.11	1.12	Overall Assessment
Assendelft, 1997 [47]	Y	Y	Y	U	U	N/A	Y	Y	Y	Y	Y	Y	Unacceptable (−)
Frymoyer, 1986 [36]	Y	Y	Y	Y	N/A	N/A	U	U	U	U	Y	N	Unacceptable (−)
Rosok, 1993 [39]	N	Y	U	N/A	Y	N	N/A	U	Y	U	Y	N	Unacceptable (−)
Haas, 1992 [40]^a^	CS	CS	CS	CS	CS	CS	CS	CS	CS	CS	CS	CS	Unacceptable (−)
Haas, 1992 [41]^a^	CS	CS	CS	CS	CS	CS	CS	CS	CS	CS	CS	CS	Unacceptable (−)
Plaugher, 1990 [48]	N	U	U	U	U	N/A	Y	Y	U	U	Y	N	Unacceptable (−)
Phillips, 1986 [43]	Y	Y	Y	Y	N/A	N/A	U	U	Y	N/A	Y	N	Unacceptable (−)
Janik, 2001 [49]	Y	Y	U	U	U	N/A	Y	Y	Y	Y	Y	Y	Unacceptable (−)
Haas, 1990 [50]	Y	Y	Y	Y	N/A	N/A	Y	Y	U	N/A	Rater 1 & 2: Y Rater 3: N	Y	Rater 1 & 2: Acceptable (+) Rater 3: Unacceptable (−)
Troyanovich, 2000 [51]	Y	Y	Y	Y	Y	N/A	Y	Y	Y	U	Y	Y	Acceptable (+)
Troyanovich, 1998 [52]	Y	Y	Y	Y	Y	N/A	Y	Y	Y	U	Y	Y	Acceptable (+)
Troyanovich, 1995 [53]	Y	Y	Y	Y	Y	N/A	Y	Y	Y	U	Y	Y	Acceptable (+)
McGregor, 1995 [46]	Y	Y	Y	Y	Y	N/A	Y	Y	N	Y	Y	Y	Acceptable (+)
Harrison, 2002 [54]^b^	Y	Y	Y	Y	Y	N/A	Y	U	Y	Y	Y	Y	Acceptable (+)
Troyanovich, 1999 [55]	Y	Y	Y	U	U	N/A	U	Y	U	U	Y	Y	Acceptable (+)
Jackson, 1993 [56]	Y	Y	Y	Y	Y	N/A	Y	Y	Y	Y	Y	Y	Acceptable (+)

*Y* yes; *N* no; *N/A* not applicable; *U* unclear; ++ high quality; + acceptable quality; − unacceptable quality/rejected

^a^No details about the methodology for the reliability study were reported in the paper

^b^ Re-calculation on data from previous study, calculations reported in evidence table with original study

Legend: Reliability Studies, 1.1 Research Question, 1.2 Representative sample, 1.3 Representative raters, 1.4 Rater blinded to other raters, 1.5 Rater blinded to own findings, 1.6 Rater blinded to reference standard, 1.7 Rater blinded to clinical information, 1.8 Rater blinded to additional cues, 1.9 Order of examination, 1.10 Time interval between measurements, 1.11 Test application and interpretation, 1.12 Appropriate statistical measures

### Data extraction

The lead author (MC) extracted data from acceptable quality (low risk of bias) studies and built evidence tables stratified by study type (Tables 3 and 4). Data extraction of each study was validated by one of four reviewers (PC, CC, SM, VK) to ensure accuracy. We contacted the study authors when clarification or additional information/data was necessary to build the evidence tables [46]. Evidence tables summarized the pertinent information and were used to create summary statements describing the body of evidence.

**Table 3:** Evidence Tables

Diagnostic Studies					
Author(s), Year	Design, Sample size (n)	Case definition	Index test	Reference Standard	Validity
McAviney, 2005 [45]	Study of criterion validity (Phase 1 for AWB and Phase 2 for ARA) *n* = 277	Cervical x-rays from randomly selected patients from Summer Hill Chiropractic Outpatient Clinic (Macquarie University, Australia), over 7 years Exclusion: moderate to severe degenerative changes; cervical spine with obvious lordosis and kyphosis; history of trauma.	Sagittal cervical alignment on x-ray films using posterior tangent method: ARA of cervical lordosis from C2-C7, AWB of the head (horizontal distance of posterior superior body of C2 compared to vertical line from posterior inferior body of C7) Partitioned into categories with increments of 5°	Presence/Absence of cervical complaints: patients’ records, history in intern’s radiology report and x-ray referral slip	ARA: Cervical complaint: 9.6° Non-cervical complaint: 23.4° ARA < 20° (to identify cervical complaint) Sn: 0.724 Sp: 0.737 AUC: 0.803 AWB: Cervical complaint: 21.3 mm Non-cervical complaint: 21.1 mm NS difference between groups
McGregor, 1995 [46]	Phase 2 study *n* = 512	New patients, > 18 YO, Canadian Memorial Chiropractic College outpatient clinic, neck and/or head pain, excluding patients diagnosed with pathology Asymptomatic subjects from small normative group from a different study Assessed for intersegmental clinical hypermobility: mobility of a given motion unit in the cervical spine which is excessive and is accompanied by local and/or peripheral symptoms	AP, lateral, AP open-mouth, forward flexion and extension cervical radiographs Including history and physical examination findings summarized in a standardized case report form	AP, lateral, AP open-mouth cervical radiographs Including history and physical examination findings summarized in a standardized case report form	With flexion-extension radiographs (3 raters): Sn: 0.65–0.89 Sp: 0.49–0.92 Without flexion-extension radiographs (3 raters): Sn: 0.11–0.91 Sp: 0.64–0.99

*AP* anteroposterior; *ARA* absolute rotation angle; *AWB* anterior weight bearing; *DDD* degenerative disc disease; *DJD* degenerative joint disease; *LR+* positive likelihood ratio; *LR-* negative likelihood ratio; *NS* no significant; *PPV* positive predictive value; *NPV* negative predictive value; *ROC* receiver operating characteristic; *Sn* Sensitivity; *Sp* Specificity; *VAS* visual analog scale

**Table 4:** Evidence Tables

Reliability Studies				
Author(s), Year	Design, Sample size (n)	Sample description	Measurement method	Measure of Reliability
Troyanovich, 2000 [51]	Intra-rater and inter-rater reliability; 3 chiropractors familiar with Chiropractic BioPhysics® technique of measurement *n* = 36 antero-posterior cervical spine radiographs	Digitized AP cervical spine radiographs without artifacts or other obvious identifying features with the second cervical vertebra through the fourth thoracic vertebra clearly depicted; from patient files of a private chiropractic office	2-dimensional coordinates of 30 points selected by each examiner: R and L narrow-waisted-appearing area of vertebral bodies T1-T4, R and L narrow-waisted-appearing area of the articular pillars of C3-C7, inferolateral aspect of both superior articular facets of C2, most superior portion of spinous process of C2-T4	**Intra-rater reliability:** **ICC (95% CI), SEM** T_x_ Rater 1: 0.99 (0.98–0.99), 1.53 Rater 2: 0.99 (0.99–1.00), 1.03 Rater 3: 1.00 (0.99–1.00), 0.99 Vertebra_apex_: Rater 1: 0.96 (0.93–0.98), 0.99 Rater 2: 0.96 (0.92–0.98), 1.10 Rater 3: 0.97 (0.94–0.98), 0.93 Rz: Rater 1: 0.97 (0.94–0.99), 1.13 Rater 2: 0.94 (0.89–0.97), 1.64 Rater 3: 0.98 (0.95–0.99), 1.06 CDA: Rater 1: 0.95 (0.91–0.97), 1.52 Rater 2: 0.92 (0.84–0.96), 2.12 Rater 3: 0.94 (0.88–0.97), 1.80 **Crossed ICC (95% CI)**^**a**^ [54] CDA: 0.93 (0.88–0.96) Rz^T1-T4^:0.96 (0.94–0.98) Tx^apex^: 0.96 (0.93–0.98) Tx^C2-T4^: 0.99 (0.99–1.00) **Interrater reliability:** **ICC (95% CI)** T_x_: 0.99 (0.99–1.00), 1.12 Vertebra_apex_: 0.98 (0.96–0.99), 0.80 Rz: 0.98 (0.97–0.99), 0.85 CDA: 0.97 (0.95–0.98), 1.22 **Crossed ICC (95% CI)**^**a**^ Cervical Spine CDA: 0.91 (0.85–0.94) Rz^T1-T4^: 0.95 (0.90–0.96) Tx^apex^: 0.93 (0.90–0.96) Tx^C2-T4^: 0.99 (0.98–0.99)
Troyanovich, 1998 [52]	Intra-rater and Inter-rater reliability; 3 chiropractors certified in use of Chiropractic Biophysics® measurement analysis *n* = 50, lateral lumbar radiographs	Lateral lumbar radiographs without artifacts or other identifying features; from patient files of a private chiropractic office	1 rater: CBP® standard manual method line drawing of radiographs 2 raters: CBP® standard method of analysis using computerized radiographic digitizer Measurements derived from 17 selected points used to construct following: ARA, ARCU, FERG, COBB, S(z), RRAs	**Intra-rater reliability** Rater 1 ICC (95% CI); SEM) T12-L1: 0.54 (0.31–0.71); 2.16 L1-L2: 0.75 (0.60–0.85); 1.82 L2-L3: 0.77 (0.63–0.87); 1.44 L3-L4: 0.85 (0.75–0.91); 1.33 L4-L5: 0.93 (0.88–0.96); 1.39 L5-S1: 0.95 (0.92–0.97); 1.68 ARA: 0.97 (0.94–0.98); 1.74 ARCU: 0.99 (0.99–1.00); 0.74 FERG: 0.94 (0.89–0.96); 1.83 COBB: 0.89 (0.81–0.94); 3.07 Sx: 1.00 (1.00–1.00); 1.07 Rater 2 ICC (95% CI); SEM) T12-L1: 0.70 (0.53–0.82); 1.46 L1-L2: 0.78 (0.64–0.87); 1.43 L2-L3: 0.61 (0.40–0.76); 2.30 L3-L4: 0.66 (0.47–0.79); 2.20 L4-L5: 0.92 (0.87–0.95); 1.44 L5-S1: 0.96 (0.94–0.98); 1.49 ARA: 0.98 (0.96–0.99); 1.47 ARCU: 0.93 (0.87–0.96); 2.40 FERG: 0.84 (0.73–0.90); 2.85 COBB: 0.88 (0.79–0.93); 3.32 Sx: 0.98 (0.97–0.99); 2.89 Rater 3 ICC (95% CI); SEM) T12-L1: 0.76 (0.61–0.86); 1.36 L1-L2: 0.77 (0.63–0.86); 1.48 L2-L3: 0.71 (0.54–0.82); 1.73 L3-L4: 0.70 (0.52–0.82); 1.77 L4-L5: 0.91 (0.85–0.95); 1.40 L5-S1: 0.97 (0.95–0.98); 1.40 ARA: 0.96 (0.93–0.98); 1.88 ARCU: 0.87 (0.78–0.92); 3.40 FERG: 0.83 (0.73–0.90); 2.77 COBB: 0.95 (0.92–0.97); 1.99 Sx: 0.99 (0.98–0.99); 2.14 **Inter-rater reliability** Rater 1–2 (manual-computer) ICC (95% CI); SEM ARA L1–5: 0.98 (0.96,0.99); 1.40 ARCU: 0.97 (0.95–0.98); 1.48 FERG: 0.88 (0.80–0.93); 2.42 COBB: 0.88 (0.79–0.93); 3.22 S(z): 0.99 (0.99–1.00); 1.70 RRAs: T12-L1: 0.68 (0.50–0.81); 1.49 L1-L2: 0.79 (0.65–0.87); 1.45 L2-L3: 0.77 (0.63–0.86); 1.49 L3-L4: 0.83 (0.71–0.90); 1.40 L4-L5: 0.90 (0.84–0.94); 1.56 L5-S1: 0.97 (0.94–9.98); 1.42 Rater 1–3 (manual-computer) ICC (95% CI); SEM ARA L1–5: 0.96 (0.93,0.98); 1.94 ARCU: 0.85 (0.76,0.91); 3.32 FERG: 0.79 (0.65,0.87); 3.25 COBB: 0.83 (0.72,0.90); 3.78 S(z): 1.00 (0.99,1.00); 1.36 RRAs: T12-L1: 0.66 (0.47,0.79); 1.59 L1-L2: 0.74 (0.58,0.84); 1.62 L2-L3: 0.76 (0.61,0.85); 1.43 L3-L4: 0.78 (0.65,0.87); 1.46 L4-L5: 0.88 (0.81,0.93); 1.64 L5-S1: 0.80 (0.67,0.88); 3.61 Rater 2–3 (computer-computer) ICC (95% CI); SEM ARA L1–5: 0.96 (0.94,0.98); 1.76 ARCU: 0.83 (0.73,0.90); 3.60 FERG: 0.84 (0.74,0.91); 2.63 COBB: 0.92 (0.86,0.95); 2.67 S(z): 0.99 (0.98,0.99); 2.16 RRAs: T12-L1: 0.63 (0.43,0.77); 1.57 L1-L2: 0.72 (0.55,0.83); 1.53 L2-L3: 0.72 (0.55,0.83); 1.67 L3-L4: 0.72 (0.55,0.83); 1.70 L4-L5: 0.90 (0.84,0.94); 3.50 L5-S1: 0.81 (0.70,0.89); 3.50
Troyanovich, 1995 [53]	Intra-rater and inter-rater reliability; 3 chiropractors certified in Chiropractic BioPhysics® method of measurement *n* = 35 lateral lumbar radiographs	Lateral lumbar radiographs without artifacts or other obvious identifying features selected from patient files of a private, primary-care chiropractic clinic	Arcuate line, Ferguson’s sacral-base line, vertical axis line, L1 and L5 stress lines and L1 and L5 posterior body lines, arcuate angle, relative rotation angle, absolute rotation angle, linear anterior or posterior displacement of the lower thoracic spine	**Intra-rater reliability:** **ICC (95% CI), SEM** L1-L5 Rater 1: 0.98 (0.92–0.99), 1.48 Rater 2: 0.98 (0.95–0.99), 1.53 Rater 3: 0.98 (0.96–0.99), 1.58 Sz Rater 1: 0.99 (0.99–1.00), 1.86 Rater 2: 0.97 (0.94–0.98), 4.26 Rater 3: 0.99 (0.98–1.00), 1.97 AA Rater 1: 0.40 (0.02–0.65), 5.03 Rater 2: 0.81 (0.65–0.90), 2.93 Rater 3: 0.71 (0.49–0.85), 3.53 FERG Rater 1: 0.97 (0.94–0.98), 1.41 Rater 2: 0.97 (0.94–0.98), 1.45 Rater 3: 0.91 (0.82, 0.95), 2.12 L1-L2 Rater 1: 0.87 (0.76, 0.93), 1.77 Rater 2: 0.84 (0.71–0.92), 1.84 Rater 3: 0.94 (0.88–0.97), 1.3 L2-L3 Rater 1: 0.85 (0.72–0.92), 1.54 Rater 2: 0.81 (0.66–0.90), 1.31 Rater 3: 0.80 (0.64–0.89), 1.79 L3-L4 Rater 1: 0.89 (0.79–0.94), 1.09 Rater 2: 0.81 (0.66–0.90), 1.52 Rater 3: 0.78 (0.60–0.88), 1.67 L4-L5 Rater 1: 0.89 (0.80–0.94), 1.49 Rater 2: 0.92 (0.85–0.96), 1.17 Rater 3: 0.87 (0.76–0.93), 1.69 **Inter-rater reliability:** **ICC (95%), SEM** L1-L5: 0.98 (0.96–0.99), 1.66 Sz: 0.98 (0.97–0.99), 3.20 AA: 0.66 (0.48, 0.79), 3.51 FERG: 0.95 (0.91–0.97), 1.73 L1-L2: 0.88 (0.81–0.94), 1.63 L2-L3: 0.84 (0.74–0.91), 1.43 L3-L4: 0.91 (0.85, 0.95), 0.97 L4-L5: 0.93 (0.89–0.96), 1.14
Haas, 1990 [50]	Inter-rater reliability; 2 radiology residents *n* = 58	PA, PA right and left lateral bending lumbar radiographs of volunteer students in a chiropractic institution	Vertebral body rotation and vertebral body tilting (intersegmental tilt measured as neutral, L or R lateral bending), radiographs categorized into: I. Ipsilateral tilt with contralateral rotation II. Ipsilateral tilt with ipsilateral rotation III. Contralateral tilt with contralateral rotation IV. Contralateral tilt with ipsilateral rotation	**L Lateral Bending Radiograph** **Global Motion: K (SE)** **V = overall agreement** Rater 1 and 2 I. 0.63 (0.17) II. 0.60 (0.17) III. 0.54 (0.17) IV. 0.71 (0.17) V. 0.60 (0.10) **R Lateral Bending Radiograph** **Global Motion: K (SE)** **V = overall agreement** Rater 1 and 2 I. 0.64 (0.17) II. 0.61 (0.16) III. 0.09 (0.17) IV. 0.72 (0.16) V. 0.58 (0.10)
McGregor, 1995 [46]	Intrarater and interrater reliability; 2 chiropractic radiology residents *n* = 506	Neutral lateral, flexion lateral and extension lateral radiographs	Measure intersegmental motion excursion of each vertebra (% of sagittal body diameter)	**Intrarater reliability: Generalizability coefficients** C2 Flexion: 0.47 C2 Extension: 0.53 C3 Flexion: 0.66 C3 Extension: 0.68 C4 Flexion: 0.67 C4 Extension: 0.74 C5 Flexion: 0.56 C5 Extension: 0.74 C6 Flexion: 0.65 C6 Extension: 0.59 C7 Flexion: 0.49 C7 Extension 0.07 **Interrater reliability: Generalizability coefficients** C2 Flexion: 0.36–0.43 C2 Extension: 0.35–0.43 C3 Flexion: 0.60 C3 Extension: 0.67 C4 Flexion: 0.63 C4 Extension: 0.70–0.77 C5 Flexion: 0.55–0.56 C5 Extension: 0.70–0.71 C6 Flexion: 0.53–0.58 C6 Extension: 0.50–0.53 C7 Flexion: 0.02 C7 Extension 0.00
Troyanovich, 1999 [55]	Intra-rater and inter-rater reliability; 3 chiropractors familiar with Chiropractic BioPhysics® technique method of measurement *n* = 37 anterioposterior lumbopelvic radiographs	Digitized AP lumbopelvic radiographs without artifacts or other obvious identifying features	2-dimensional coordinates of 33 points selected by each examiner: R and L superior and inferior corners of each vertebral body from T12 through L5, the most superior portion of the spinous processes of T12 through L5 and S2, and the R and L superolateral aspects of the sacral base Computer calculated lines of lateral displacement from true vertical, magnitude of angle of intersection of two lines (LDA), angle of intersection of inferior line with sacral base line (LS angle), horizontal line across sacral base (HB line), true vertical axis line from the spinous process of S2 cephalically and parallel to the lateral edge of the x-ray film (VAL)	**Intra-rater reliability** **ICC (95% CI), SEM** HB angle Rater 1: 0.72 (0.52–0.84), 1.62 Rater 2: 0.75 (0.57–0.87), 1.78 Rater 3: 0.94 (0.89–0.97), 0.67 LD angle Rater 1: 0.91 (0.83–0.95), 1.22 Rater 2: 0.90 (0.82–0.95), 1.33 Rater 3: 0.96 (0.92–0.98), 0.87 LS angle Rater 1: 0.84 (0.72–0.92), 2.04 Rater 2: 0.88 (0.77–0.93), 2.07 Rater 3: 0.96 (0.93–0.98), 0.93 Tx^T12^ Rater 1: 0.97 (0.94–0.98), 1.53 Rater 2: 0.95 (0.91–0.97), 1.95 Rater 3: 0.97 (0.95–0.99), 1.40 **Crossed ICC (95% CI)**^**a**^ HB angle: 0.78 (0.67–0.86) LD angle: 0.92 (0.87–0.95) LS angle: 0.88 (0.81–0.93) Tx_T12-S1_: 0.96 (0.94–0.98) **Inter-rater reliability** **ICC (95% CI), SEM** HB angle: 0.71 (0.56–0.82), 1.62 LD angle: 0.97 (0.94–0.98), 0.75 LS angle: 0.83 (0.73–0.90), 2.13 Tx^T12^: 0.95 (0.91–0.97), 2.01 **Crossed ICC (95% CI)**^**a**^ [54] HB angle: 0.61 (0.49–0.73) LD angle: 0.89 (0.83–0.94) LS angle: 0.76 (0.66–0.85) Tx_T12-S1_: 0.92 (0.88–0.95)
Jackson, 1993 [56]	Intrarater and interrater reliability; 3 chiropractors certified in use of Chiropractic BioPhysics® *n* = 65	Lateral cervical films from patient files of a primary care private chiropractic clinic	Standard CBP® measurement protocols: Atlas plane line, Ruth Jackson’s stress lines, vertical axis line and C2 through C7 posterior body lines; relative rotation angle measurements, ARA	**Intra-rater reliability** Not reported due to inadequate statistics used to compute reliability. **Inter-rater reliability** **Bartko’s ICC; SEM** Atlas plane line: 0.93; 1° ARA: 0.96; 1.20° Anterior head translation: 0.80; 1.23 mm Intersegmental angle C2–3: 0.72; 0.57° Intersegmental angle C3–4: 0.79; 0.54° Intersegmental angle C4–5: 0.86; 1.04° Intersegmental angle C5–6: 0.79; 0.66° Intersegmental angle C6–7: 0.74; 0.65°

*AA* arcuate angle; *ARA* absolute rotation angle; *ARCU* arcuate angle measurement; *CBP®* Chiropractic Biophysics®; *CDA* cervicodorsal angle; *COBB* Cobb angle measurement; *FERG* Ferguson’s angle measurement; *HB angle* angle of sacral base compared to horizontal; *HB line* horizontal line intersection line drawn across the sacral base; *L* left; *LD angle* lumbo-dorsal angle, angle of best fit line form lumbar apex to L5 compared to the sacral base; *LS angle* lumbosacral angle, angle of best fit line from lumbar apex to L5 compared to the sacral base; *R* right; *RRA* intersegmental measurements; *Ry* segmental axial rotation angles; *Rz* magnitude of the angle of intersection between vertical axis line and lower most line; *SEM* standard error of measurement; *S(z)* translation measurement of lower thoracic spine to S1; *T*_*x*_ perpendicular distance from vertical axis line to the center of the vertebral body of C2; *Tx*^*T12*^ lateral translation distance of T12 compared to 9S2; *VAL* vertical axis line; vertebra_apex_: linear distance from center of vertebra most displaced from line connecting the centers of C2 and T4

^a^ Harrison 2002 [54] calculated crossed ICCs from two individual studies, these calculations are presented with the original articles

### Data items

Information extracted from each diagnostic study included the study design, sample population, case definition, index test, reference standard and results of the study. Information extracted from each reliability study included study design, sample size, sample description, measurement method and results of the study.

### Statistical analyses

When data were available, we computed the measurement mean change (and 95% confidence intervals) from diagnostic studies. Confidence intervals (CI) were calculated using mean change in each group, standard deviation, total number of participants in each group, and α = 0.05.

### Evidence synthesis

We used the best evidence synthesis methodology to conduct a qualitative synthesis of the evidence from acceptable quality (low risk of bias) studies [57, 58]. The evidence synthesis provides conclusions based on the best available evidence or may conclude that there is insufficient evidence to make any conclusions [57].

We stratified diagnostic studies into one of four phases, as described by Sackett [28]. Phase one studies test results in patients with the target condition compared to those without the target condition [28]. Phase two studies test whether patients with certain test results are more likely to have the target disorder than patients with differing results [28]. Phase three studies determine whether test results distinguish patients with and without the target disorder among patients in whom it is clinically reasonable to suspect the disease is present [28]. Phase four studies determine whether patients who undergo the test have improved health outcomes compared to similar patients who are not tested [28].

### Reporting of outcomes

If we retrieved relevant RCTs, we aimed to check the clinical trials registry (Clinicaltrials.gov) to assess for outcome reporting bias.

## Results

### Study selection

**Fig. 2:**
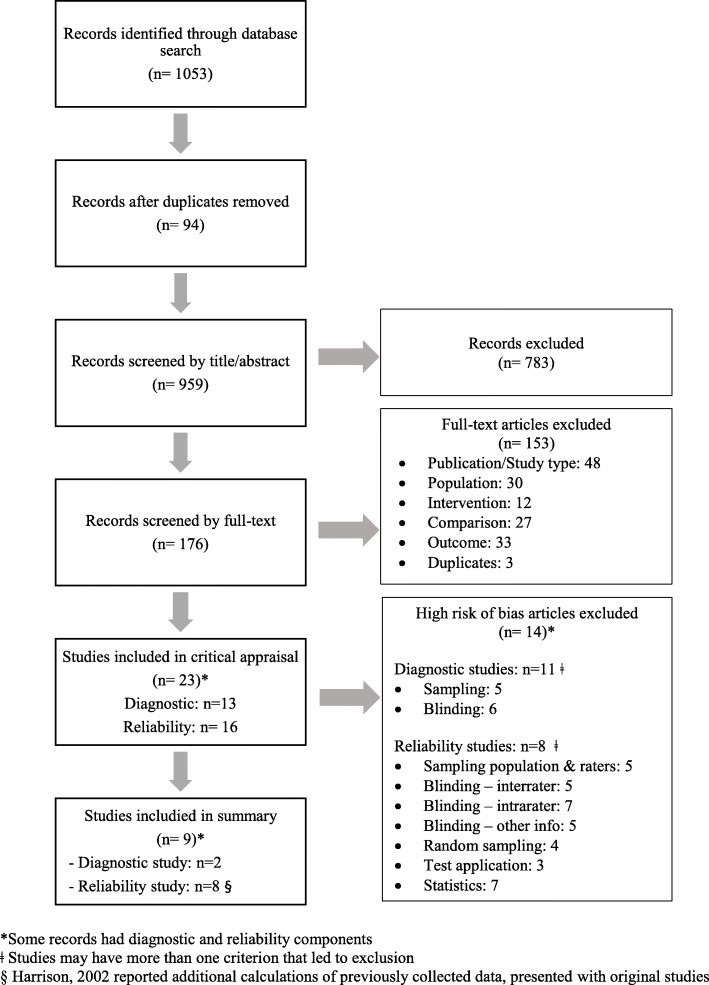
Flow diagram of study selection

Our search retrieved 1053 citations (Fig. 2). We removed 94 duplicates and screened 959 articles. Inter-rater agreement for phase one screening was 95.8% between MC and CC. We screened 176 full-text articles (phase two). Inter-rater agreement for phase two screening was 95.4% between MC and CC. Of those, 23 articles met the inclusion criteria and were eligible for critical appraisal. Reasons for exclusion were ineligible publication type (*n* = 48), population not including patients presenting to chiropractors in the absence of red flags (*n* = 30), intervention did not include spinal radiographs (*n* = 12), did not have a comparison group (*n* = 27), outcomes were not structural or functional findings on radiographs (*n* = 33) and duplicates (*n* = 3).

### Risk of Bias

We found no relevant studies investigating the diagnostic or therapeutic utility of routine or repeat radiographs for structural or functional evaluation of the spine. Similarly, we found no studies investigating the use of repeat radiographs for functional or structural findings of the spine to monitor clinically meaningful changes in conditions or care for patients (Fig. 2).

We critically appraised 23 studies investigating the validity or reliability of radiographs for the functional or structural evaluation of the spine. Of these, 14 were at risk of bias and excluded from the best evidence synthesis [34–44, 47–49]. These included 11 diagnostic studies and eight reliability studies (six of the 11 studies had both diagnostic and reliability components). The diagnostic studies with a risk of bias had methodological limitations including 1) inadequate population sampling (*n* = 5), and 2) inadequate blinding (*n* = 6). In the reliability studies with a risk of bias, methodological limitations included: 1) poor population and/or rater sampling (*n* = 5), 2) inadequate inter-rater, intra-rater or information blinding (*n* = 17), 3) no random sampling (*n* = 4) and 4) poor test application and interpretation (*n* = 3) (Tables 1 and 2). We did not identify any cohort, case-control, or cross-sectional studies. Additionally, we did not identify any RCTs, therefore we did not check the clinical trials registry.

We included nine low risk of bias studies in our best evidence synthesis; one diagnostic study [45], seven reliability studies [50–56] and one study with diagnostic and reliability components [46]. One reliability study provided further analyses to previously collected data, which were reported with the original studies [54]. These studies had some methodological limitations, but not in sampling, blinding or random sampling (Tables 1 and 2).

### Study characteristics

We included eight reliability studies, [46, 50–53, 55, 56] five that examined the intra- and inter-rater reliability of Chiropractic BioPhysics®, of which four that investigated cervical spine measurements [51, 53, 55, 56] and one that studied lumbar spine measurements [52]. One study examined the intra- and inter-rater reliability of flexion-extension radiographs in addition to a standard cervical radiograph series, [46] and one investigated the inter-rater reliability of vertebral rotation and tilt of lateral bending radiographs [50]. We included two phase two diagnostic (validity) studies, [45, 46] that investigated whether patients with radiographic findings were more likely to have the target disorder than patients with other test outcomes [28]. One study investigated radiographic findings of spinal degeneration and cervical complaints [45] and the other investigated findings on flexion-extension radiographs of intersegmental clinical hypermobility [46].

### Reliability of radiographic measurements

Four studies investigating Chiropractic BioPhysics® measurements of the cervical spine (i.e., anterior head translation, vertebral translation in the cervical and thoracic spine, cervical lordosis angle, cervicodorsal angle, absolute rotation angle, Ferguson’s angle, Cobb angle and intersegmental measurements) found that these were performed with acceptable levels of reliability (Tables 3 and 4) [51, 53, 55, 56]. One study investigated Chiropractic BioPhysics® measurements of the lumbar spine (i.e., sacral base angle, lumbodorsal angle, lumbosacral angle and lumbar spine vertebral translation) also reported acceptable levels of reliability [52]. The one exception was the measurement of the arcuate angle, which had a low to acceptable level of reliability in the cervical and lumbar spine [52, 53].

For other radiographic measurements, Haas et al. found that categorizing vertebral body rotation and tilting into five categories, may be associated with poor reliability and significant measurement error [50]. Similarly, McGregor et al. reported that measuring intersegmental motion of each vertebra in flexion and extension is associated with poor reliability and significant measurement error [46].

### Validity of radiographic measurements

We did not identify any studies of acceptable methodological quality providing evidence of the diagnostic accuracy (sensitivity, specificity, predictive values) of Chiropractic BioPhysics® measurements. Thus, we do not know if these measurements are evaluating clinically important outcomes for conditions of the cervical or lumbar spine.

Two low risk of bias studies provided preliminary evidence, phase two diagnostic studies, of the diagnostic validity of using radiographs for functional and structural evaluation of the spine [45, 46]. McAviney et al. [45] investigated the association of cervical radiograph measurements in patients with and without cervical spine complaints. The authors did not find significant differences in head anterior weight bearing between participants with or without cervical complaints [45]. However, they reported that participants with less than 20° of absolute rotation angle (a measure of cervical lordosis) were greater than two times more likely to have cervical complaints compared to those who had more than 20° [45]. McGregor et al. [46] investigated the benefit of adding cervical flexion-extension radiographs to a normal series of cervical radiographs and standardized case report for the diagnosis of intersegmental clinical hypermobility. They reported no additional diagnostic benefit of using flexion-extension radiographs [46].

### Clinical utility

We did not identify any relevant studies investigating the diagnostic or therapeutic utility of cervical, thoracic or lumbar radiographs (in the absence of red flags) for the functional or structural evaluation of the spine. Similarly, we did not identify any relevant studies that investigated whether functional or structural findings on repeat radiographs of the cervical, thoracic or lumbar spine are valid markers of clinically meaningful change when monitoring conditions or managing patients.

## Discussion

Clinical utility refers to the degree to which the use of a test (such as radiographs) is associated with changing health outcomes through diagnosis or selection of an appropriate treatment [17–19]. We did not find evidence that cervical, thoracic or lumbar radiographs (in the absence of red flags) obtained for the purpose of evaluating the function or structure evaluation of the spine can benefit patients. Therefore, we do not recommend that routine, or repeat radiographs of the cervical, thoracic or lumbar spine (in the absence of red flags) be used by chiropractors to evaluate the structure or function of the spine for diagnostic or therapeutic purposes.

Although we found eight reliability studies and two diagnostic (phase two) validity studies with a low risk of bias, these studies cannot be used to justify using routine or repeat radiographs of the spine [45, 46, 50–56]. While some measurements of cervical and lumbar spine radiographs have acceptable levels of reliability, and preliminary evidence of diagnostic validity, we did not identify any acceptable studies investigating their clinical utility.

Several evidence-based clinical practice guidelines are available to inform the use of radiographs in cases of trauma, or when pathology is suspected [3–5, 7]. Moreover, guidelines make clear recommendations against the use of radiographs to assess function of the spine [5, 7]. While our rapid review agrees with these statements, it nevertheless conflicts with recommendations published by the International Chiropractic Association in the document entitled: “*Practicing Chiropractors’ Committee on Radiology Protocols (PCCRP) for Biomechanical Assessment of Spinal Subluxation in Chiropractic Clinical Practice*”, a guideline frequently referenced by a subset of chiropractors [13]. The divergent conclusions are attributable to differences in methodology, in particular differences in the search strategy and selection of articles. The development of the PCCRP document did not include a risk of bias assessment of eligible studies. Thus the synthesis included low quality studies which likely biased the recommendations made by that guideline expert panel [59]. Furthermore, it is unclear whether the guideline expert panel had editorial independence; most members (17/25) of the guideline expert panel and investigators were members of the sponsoring organization [13].

In a review by Triano et al. [60], they used a consensus process to assess the appropriateness of imaging as a diagnostic tool to guide the use of manual therapy. Despite the low quality and narrative nature of their review, the use of radiographs to localize the site of care for manual therapy was not recommended. However, contrary to our findings, they recommended the use of static and motion radiographic studies to identify hypermobile but not hypomobile segments. Our study included two relevant low risk of bias studies [46, 50] suggesting poor reliability of radiographs to assess motion patterns, and one preliminary phase two diagnostic study [46] that was not included in their review, that clearly contradicts their recommendation.

We live in the era of value-based health care [61]. One of the goals of value-based healthcare is to reduce the utilization of low-value tests and interventions that do not benefit patients but increase the costs of care. Campaigns such as Choosing Wisely® have been designed and implemented to promote conversations between clinicians and patients by helping patients choose care that is: 1) supported by evidence; 2) not duplicative of other tests or procedures already received; 3) free from harm; and 4) truly necessary [62]. In 2017, the American Chiropractic Association adapted the Choosing Wisely® recommendations on lumbar spine radiography [63] and recommended to avoid routine spinal imaging in the absence of clear clinical indicators for patients with acute low back pain of less than 6 weeks duration. Furthermore, the American Chiropractic Association recommended that repeat imaging must not be used to monitor patients’ progress [62]. Our findings are in agreement with the American Chiropractic Association adapted Choosing Wisely® recommendations.

A principle of value-based health care is that clinical interventions should be free from harm, or at the very least, the benefits of the intervention must substantially outweigh the risks [63]. A known risk for ionizing exposure is the increased frequency of cancer beyond that occurring spontaneously and non-cancer diseases (i.e. cardiovascular diseases) [64–66]. Studies have shown that 100 mSv is the approximate dose of radiation to be received by a patient before there is a known increased risk of cancer over a lifetime [64, 67, 68]. The current widely used theory on radiation accumulation is based on the linear no-threshold (LNT) model which in simple terms states: no dose of radiation exists without risk and that risk increases proportionally with dose [68, 69]. Currently, direct risks associated with low doses, as those received with radiographic studies, in the LNT model are unknown [64–66].

However, despite the ongoing debate of the LNT theory, [70, 71] the argument remains that radiographic studies should not be considered in isolation, but viewed as part of the patient’s lifetime exposure. Ionizing radiation is a cumulative process that occurs from natural sources, such as sunlight, and decay of elements in our environment, as well as man-made sources, such as medical imaging (i.e. radiographs, computed tomography (CT) and nuclear medicine scans) [62, 63]. It is therefore recommended by the International Commission on Radiological Protection (ICRP) and the Canadian Nuclear Safety Commission (CNSC), that in the absence of information pertaining to low-dose risks, to follow the “as low as reasonably achievable” (ALARA) principle [64]. ALARA is not a dose limit, but a practice that aims to keep the dose levels as far as possible below the regulatory limit [64, 72]. In light of the inherent risks of the use of ionizing radiation, and given that the clinical utility is unknown, the use of routine and repeat radiographs for the purpose of assessing functional or structural evaluation of the spine is not recommended.

Our rapid review has limitations inherent to the rapid review methodology [21]. These limitations include: 1) focused search of the literature (three databases) which may lead to studies being omitted from the review; and 2) the conduct of screening, critical appraisal and data extraction done by one investigator instead of two. However, we reduced the impact of these limitations by: carefully selecting databases where the relevant literature is most likely to be published (MEDLINE, CINAHL, and ICL); and implementing a structured quality assurance methodology to minimize error in screening and selection of articles, and data extraction.

## Conclusion

Radiographs are an important diagnostic tool in patient management when clinical indicators of serious pathologies (red flags) are present. We found no evidence that radiographs used to assess the function or structure of the spine improves patients’ outcomes. Therefore, in the absence of red flags, and given the inherent risks of ionizing radiation, we do not recommend the clinical use of radiographs for the routine and repeat evaluation of the structure and function of the spine.

## Supplementary information

**Additional file 1.** MEDLINE Search Strategy.

**Additional file 2.** Glossary.

## Data Availability

The datasets used and/or analyzed during the current study are available from the corresponding author on reasonable request.

## Abbreviations

ALARA: As low as reasonably achievable
CNSC: Canadian Nuclear Safety Commission
ICRP: International Commission on Radiological Protection
RCT: Randomized controlled trials
