# Supplementary material for: The clinical utility of routine spinal radiographs by chiropractors: a rapid review of the literature
Source: Chiropr Man Therap. 2020 Jul 9;28:33. doi: 10.1186/s12998-020-00323-8 (PMC7346665; doi:10.1186/s12998-020-00323-8)
Supplement: Supplementary file 1 — Additional file 1. MEDLINE Search Strategy. [file 12998_2020_323_MOESM1_ESM.docx]

**APPENDIX 1: MEDLINE Search Strategy**

Search run November 25, 2019 in Ovid MEDLINE: Epub Ahead of Print, In-Process & Other Non-Indexed Citations, Ovid MEDLINE® Daily and Ovid MEDLINE® 1946-Present; 691 results

| 1 | exp Whiplash Injuries/ | 3199 |
| --- | --- | --- |
| 2 | exp Neck Injuries/ | 7871 |
| 3 | exp Neck Pain/ | 6711 |
| 4 | Neck Muscles/in [Injuries] | 150 |
| 5 | exp Cervical Vertebrae/in [Injuries] | 7798 |
| 6 | exp Radiculopathy/ | 4927 |
| 7 | exp Brachial Plexus Neuropathies/ | 3644 |
| 8 | exp Torticollis/ | 3509 |
| 9 | whiplash.ab,ti. | 2993 |
| 10 | "neck injur*".ab,ti. | 1778 |
| 11 | "neck pain*".ab,ti. | 9100 |
| 12 | "cervical pain*".ab,ti. | 978 |
| 13 | "neck ache*".ab,ti. | 31 |
| 14 | "neckache*".ab,ti. | 23 |
| 15 | "cervicalgia*".ab,ti. | 122 |
| 16 | "cervicodynia*".ab,ti. | 9 |
| 17 | "radiculopath*".ab,ti. | 6020 |
| 18 | "brachial plexus neuropath*".ab,ti. | 187 |
| 19 | torticollis.ab,ti. | 3165 |
| 20 | WAD.ab,ti. | 845 |
| 21 | NAD.ab,ti. | 33353 |
| 22 | or/1-21 [**neck pain] | 77434 |
| 23 | exp Back Injuries/ | 24002 |
| 24 | exp Back Pain/ | 37733 |
| 25 | Coccyx/in [Injuries] | 95 |
| 26 | Intervertebral Disc Degeneration/ | 4692 |
| 27 | intervertebral disk hernia/ | 0 |
| 28 | Lumbar Vertebrae/in [Injuries] | 4631 |
| 29 | exp Lumbosacral Plexus/ | 36225 |
| 30 | Lumbosacral Region/in [Injuries] | 205 |
| 31 | Osteoarthritis, Spine/ | 165 |
| 32 | Piriformis Muscle Syndrome/ | 117 |
| 33 | Polyradiculopathy/ | 2536 |
| 34 | Sacroiliac Joint/ | 3933 |
| 35 | Sacrococcygeal Region/ | 3726 |
| 36 | Sacrum/ | 8784 |
| 37 | Spinal Diseases/ | 20905 |
| 38 | Spinal Stenosis/ | 5855 |
| 39 | Sciatica/ | 4934 |
| 40 | (avulsed lumbar adj3 (disc* or disk*)).ab,ti. | 0 |
| 41 | (back adj3 (ache* or injur* or pain*)).ab,ti. | 48943 |
| 42 | (backache* adj3 (injur* or pain*)).ab,ti. | 268 |
| 43 | (back pain or back-pain).ab,ti. | 44476 |
| 44 | coccydynia.ab,ti. | 127 |
| 45 | coccyx.ab,ti. | 728 |
| 46 | dorsalgia.ab,ti. | 88 |
| 47 | (lumbar disc* adj3 (extruded or degenerat* or herniat* or prolapse* or sequestered or slipped)).ab,ti. | 4356 |
| 48 | (lumbar disk* adj3 (extruded or degenerat* or herniat* or prolapse* or sequestered or slipped)).ab,ti. | 760 |
| 49 | "low* back pain".ab,ti. | 27729 |
| 50 | "low*-back-pain*".ab,ti. | 27772 |
| 51 | (lumbar adj3 (pain or facet or nerve root* or osteoarthritis or radicul* or spinal stenosis or spondylo* or zygapophys*)).ab,ti. | 10092 |
| 52 | "lumbarsacr*".ab,ti. | 8 |
| 53 | lumboischialgia.ab,ti. | 46 |
| 54 | "lumbosacr*".ab,ti. | 10954 |
| 55 | "Piriformis syndrome*".ab,ti. | 265 |
| 56 | radiculalgia.ab,ti. | 85 |
| 57 | (sacral adj2 pain*).ab,ti. | 191 |
| 58 | (sacrococcygeal adj2 pain*).ab,ti. | 21 |
| 59 | (sacroiliac or sacro-iliac).ab,ti. | 5123 |
| 60 | "sciatic*".ab,ti. | 27837 |
| 61 | (SI adj joint).ab,ti. | 366 |
| 62 | (spinal adj2 stenos?s).ab,ti. | 5745 |
| 63 | spondylosis.ab,ti. | 3130 |
| 64 | "tailbone adj3 pain*".ab,ti. | 0 |
| 65 | "vertebrogenic adj3 pain*".ab,ti. | 0 |
| 66 | or/23-65 [**back pain] | 190441 |
| 67 | Thoracic Vertebrae/ | 20201 |
| 68 | exp Thorax/ | 35971 |
| 69 | Thoracic Injuries/ | 12494 |
| 70 | (thoracic adj4 (spine or spinal)).ab,ti. | 12666 |
| 71 | (T-spine or T-spinal).ab,ti. | 59 |
| 72 | thoracic wall.ab,ti. | 2159 |
| 73 | thoracic spine.ab,ti. | 5305 |
| 74 | "thoracic spinal pain*".ab,ti. | 20 |
| 75 | "thoracic injur*".ab,ti. | 1871 |
| 76 | thorax.ab,ti. | 18382 |
| 77 | thoracolumbar.ab,ti. | 8566 |
| 78 | or/67-77 [** thoracic pain] | 96599 |
| 79 | Spondylitis, Ankylosing/ | 14350 |
| 80 | Spondylarthritis/ | 2144 |
| 81 | Rheumatic Diseases/ | 22529 |
| 82 | Arthritis/ | 35133 |
| 83 | Arthritis, Rheumatoid/ | 97119 |
| 84 | (ankylosing and (spondylarthritis or spondylarthritides or spondylitis or spondyloarthritis)).ab,ti. | 13723 |
| 85 | (rheumatoid and (arthritis or spondyloarthritis)).ab,ti. | 103177 |
| 86 | (spondylarthritides or spondyloarthrides).ab,ti. | 53 |
| 87 | ((spine or spinal) and (arthritis or arthritides or osteoarthritis)).ab,ti. | 5074 |
| 88 | ((spine or spinal) adj4 (condition* or degenerat* or disable* or disabilit* or disease* or disorder* or osteoarthritis or pain or stenos?s)).ab,ti. | 30603 |
| 89 | axial spondyloarthritis.ab,ti. | 1156 |
| 90 | (spondyloarthropathies or spondyloarthrides).ab,ti. | 1385 |
| 91 | arthritis.ab,ti. | 167767 |
| 92 | or/79-91 [**more spinal conditions] | 264637 |
| 93 | 22 or 66 or 78 [**cervical_lumbar_thoracic] | 338856 |
| 94 | 93 or 92 [**all spinal conditions] | 578690 |
| 95 | Diagnostic Imaging/ | 40234 |
| 96 | Radiography/ | 316291 |
| 97 | exp Radiology/ | 35747 |
| 98 | X-Rays/ | 28250 |
| 99 | "bone adj scan*".ab,ti. | 0 |
| 100 | "CAT adj scan*".ab,ti. | 0 |
| 101 | imaging.ab,ti. | 760259 |
| 102 | "radiograph*".ab,ti. | 206474 |
| 103 | "radiolog*".ab,ti. | 242982 |
| 104 | (x-ray* or xray*).ab,ti. | 339988 |
| 105 | ((imaging or radiogr* or radiol* or x-ray* or xray*) adj6 (accuracy or accurate or appropriate* or beneficial or benefit* or effect or effects or effectiv* or efficacy or efficien* or essential or importan* or necessary or reduce or reduction or safe or safety or useful or utility or validity or validation or value or valuable or sensitivity or specificity)).ab,kf,ti. | 140652 |
| 106 | ((imaging or radiogr* or radiol* or x-ray* or xray*) adj6 (adverse or complication* or contraindicate* or harm* or inaccurat* or inappropriate* or misdiagnos* or misuse* or overdiagnos* or overtreat* or overuse* or overutiliz* or overutilis* or risk* or underuse* or unnecessary or unwanted)).ab,kf,ti. | 25639 |
| 107 | ((imaging or radiogr* or radiol* or x-ray* or xray*) adj6 (excessive or frequen* or repeat* or repetition* or routine* or routinely)).ab,kf,ti. | 29392 |
| 108 | ((imaging or radiogr* or radiol* or x-ray* or xray*) adj6 (false-positive* or false positive*)).ab,kf,ti. | 835 |
| 109 | ((imaging or radiogr* or radiol* or x-ray* or xray*) adj6 (alignment* or asymmetry or symmetry or biomechanical analysis or compensation or curvature or deviat* or displacement or dysfunction* or function* or measure*)).ab,kf,ti. | 140158 |
| 110 | ((imaging or radiogr* or radiol* or x-ray* or xray*) adj6 (impairment* or segmental or intersegmental or kyphosis or lordosis or malalignment* or misalignment* or misposition* or posture or rehabilit* or spinography or subluxation* or tilt)).ab,kf,ti. | 5447 |
| 111 | or/95-110 [**x-rays] | 1652654 |
| 112 | Chiropractic/ | 3202 |
| 113 | Manipulation, Chiropractic/ | 971 |
| 114 | Manipulation, Spinal/ | 1535 |
| 115 | Musculoskeletal Manipulations/ | 1675 |
| 116 | (adjust* adj3 (chiropract* or spinal or lumbar or cervical or thoracic or instrument* or tool* or electric)).ab,kf,ti. | 1697 |
| 117 | chiropract*.ab,kf,ti. | 5861 |
| 118 | (HVLA or high velocity low amplitude).ab,kf,ti. | 261 |
| 119 | (manipulat* adj3 (chiropract* or spinal or spine or low* back or joint* or lumbar or neck or thoracic or cervical or MSK or musculoskeletal or vertebr*)).ab,kf,ti. | 3905 |
| 120 | ((manipulat* or mobili?at*) adj4 instrument*).ab,kf,ti. | 748 |
| 121 | (massage adj3 therap*).ab,kf,ti. | 2002 |
| 122 | (mobili?at* adj3 (chiropract* or spinal or spine or low* back or joint* or lumbar or neck or thoracic or cervical or MSK or musculoskeletal or vertebr*)).ab,kf,ti. | 1230 |
| 123 | ((therap* or treat* or intervention* or manag*) adj3 (manual or manipulat* or mobili?at* or MSK or musculoskeletal)).ab,kf,ti. | 16483 |
| 124 | (traction adj3 (manual or passive or mechanical or non-surgical or nonsurgical)).ab,kf,ti. | 468 |
| 125 | (flexion-distraction or flexion distraction).ab,kf,ti. | 257 |
| 126 | or/112-125 [**chiropractic] | 30053 |
| 127 | 94 and 111 and 126 | 1116 |
| 128 | limit 127 to (address or case reports or clinical conference or comment or congress or editorial or government document or letter or practice guideline or "systematic review" or systematic reviews as topic) | 346 |
| 129 | 127 not 128 | 770 |
| 130 | case series.ab,kf,ti. | 69588 |
| 131 | 94 and 111 and 126 and 130 | 27 |
| 132 | 129 or 131 | 781 |
| 133 | limit 132 to english language | 691 |
