# Supplementary material for: The clinical utility of routine spinal radiographs by chiropractors: a rapid review of the literature
Source: Chiropr Man Therap. 2020 Jul 9;28:33. doi: 10.1186/s12998-020-00323-8 (PMC7346665; doi:10.1186/s12998-020-00323-8)
Supplement: Supplementary file 2 — Additional file 2. Glossary. [file 12998_2020_323_MOESM2_ESM.docx]

**Appendix II: Glossary**

According to Fletcher et al., Clinical Epidemiology The Essentials, Third Edition:

Validity: Demonstrates that a test measures what it is intended to measure.[1]

Diagnostic accuracy: Demonstrates that the results of a test correspond to the true state of the disease being measured. To determine diagnostic accuracy, one must compare the test results and the true diagnosis. Reports of diagnostic accuracy include sensitivity, specificity, predictive values, likelihood ratios and receiver operator characteristic (ROC) curve.[1]

Reliability: Determines the stability of a test when it is repeated under different conditions or by different people.[1]

Sensitivity: The proportion of people with the disease who test positive for the disease. A sensitive test will rarely miss people who have the disease.[1]

Specificity: The proportion of people without the disease who test negative for the disease. A specific test will rarely misdiagnose people without the disease as diseased.[1]

Predictive values: Determined by the sensitivity and specificity of a test and the prevalence of the disease in the population being tested. Where prevalence is defined as the proportion of people in a population with the disease, at a given time.[1]

- Positive predictive value: The more specific a test is, the better the positive predictive value will be and the more confident the clinician can be that a positive test rules in the disease.[1]
- Negative predictive value: The more sensitive a test is, the better the negative predictive value will be and the more confident the clinician can be that a negative test rules out the disease.[1]

Likelihood ratios: Summarize the same information as sensitivity and specificity and can be used to calculate the probability of disease after a positive and negative test. Likelihood ratios tell you how many times (more or less likely) a test result is to be reported in diseased compared to non-diseased people. It is the probability of a test result in people with the disease divided by the probability of the test result in people without the disease.[1]

- Positive likelihood ratio: Likelihood ratio associated with a positive test[1]
- Negative likelihood ratio: Likelihood ratio associated with a negative test[1]

Receiver operator characteristic (ROC) curve: Shows the comparison between sensitivity and specificity for a test.[1] The overall accuracy of a test can be described as the area under the ROC curve, where the larger the area, the better the test. The ROC curve is a good way of comparing different tests for the same disease.[1]

**References**

1. Fletcher RH, Fletcher SW, Wagner EH. Clinical Epidemiology The Essentials. Third Edit. Baltimore, Maryland: Williams & Wilkins; 1996.
